# Gender differences in the effects of skin hydration on electrodermal activity measurements

**DOI:** 10.2478/joeb-2025-0006

**Published:** 2025-03-24

**Authors:** Ardawan A. Youssif, Dindar S. Bari

**Affiliations:** Department of Physics, College of Science, University of Zakho, Zakho, Kurdistan region, Iraq; Scientific Research Center, University of Zakho, Zakho, Kurdistan region, Iraq

**Keywords:** Electrodermal activity, gender, hydration, skin conductance, skin potential, skin susceptance

## Abstract

Electrodermal activity (EDA) is defined as a general term for all electrical behaviors in the skin, encompassing all active and passive electrical properties that can be traced back to the skin and its appendages. EDA measurements can be impacted by various factors and conditions. A factor of effect on EDA recordings, which has not been investigated before is the gender-related differences in the effects of skin hydration on EDA signals. Hence, this study aimed to study gender-related differences in the EDA parameters under conditions of skin hydration. 30 males and 30 females participated in this study under two different conditional (normal and hydration) experiments. Three EDA parameters (tonic and phasic components) were recorded from both groups. In the hydration experiment, 0.2 gram of 0.5% KCI in a 2% agar jelly was applied to the skin of all participants to moisturize their skin. The data from both experiments were analyzed to investigate gender-related differences in the effects of skin hydration on EDA measurements. It was found that EDA measurements, in particular the tonic component were not influenced by gender-related differences under hydration conditions. However, some significant (*p*<0.05) differences were observed between males and females in certain phasic parameters in response to specific stimuli. This study suggests that skin hydration does not contribute to gender-related differences in EDA recordings. These results are probably important in EDA investigations and applications.

## Introduction

The skin is the largest organ in the body. It has a complex structure with various layers working as an interface between the body and the external environment. The skin performs many tasks from the body's thermal regulation to the physical barrier against the penetration of pathogens and many other functions.

Gender-related differences have been pointed out in various body organs, including skin. It is shown that men's skin thickness is greater than that of women, whereas the subcutaneous (layer) fat thickness of women is greater [1, 2]. Females have been reported to have a higher surface pH than males [3, 4], conversely, it is shown that males have higher sebum production and larger pore size than females [5]. Moreover, during physical exercise, males produce sweat at a larger rate than females [6, 7]. Generally, the skin of both genders shows high ionic impedance during normal conditions since the outermost layer of the skin consists of dead skin cells (keratinized tissue). However, the skin impedance is strongly reduced when the skin is hydrated either due to sweating [8] or application of a moisturizer [9, 10].

Electrodermal activity (EDA) represents variations in the electrical characteristics of the skin surface which fluctuate in response to sweat secretion controlled by the sympathetic nervous system [11]. Changes in sweat volume are triggered by central nervous system activity associated with emotional and cognitive states. The EDA waveforms are analytically processed into two basic components: tonic and phasic components. The phasic component manifests the rapid changes in EDA in response to startle-like stimuli, whereas the slower tonic component demonstrates the gradual changes in EDA associated with the passive indicators of sympathetic activity [12, 13].

EDA parameters, which are the skin conductance responses (SCRs), skin potential responses (SPRs), and/or skin susceptance responses (SSRs), skin conductance level (SCL), skin potential level (SPL), and/or skin susceptance level (SSL), are shown to be influenced by various external factors such as environmental temperature [14], humidity [15], and sound (noise) [16], and internal or physiological factors like speech activity, deep inhalations, breath holding, etc. Also, EDA signals can be influenced by technical issues like electrode-skin connection and site of measurement, and by solid or wet gel electrodes with different electrolyte compositions. Moreover, EDA measurements may also be impacted by demographic characteristics such as age differences [17], gender differences [18], and ethnic differences and heritability [11].

Skin hydration is another factor that can affect EDA measurements. In some studies, the effects of skin hydration on EDA recordings are investigated. For instance, Fowles and Robert [19], and Fowles and Schneider [20] reported that SC and SP were influenced when the skin was hydrated, in which SC was increased and the negativity of the SP was reduced. In two separate studies Garwood et al. [21, 22], investigated age-related differences in the impact of skin hydration on SC and SP for young and old men. They observed that there were significant differences between both groups concerning SP, but not for SC. Fowles and Schneider [23], in another study examined the impacts of four various electrolytes on SPL and SPRs, and found a large influence of hydration on SPL and SPRs.

A factor of effect on EDA, which to the authors’ knowledge has never been studied before, is the gender-related differences in the effects of skin hydration on EDA. In this study, we have investigated whether hydration factors have similar effects on EDA recorded in both males and females. In general, maintaining adequate skin hydration (i.e., the necessary water content in the skin cells) is essential for keeping the skin healthy. Moistening of the skin will cause changes in its electrical properties such as skin resistance [11].

The aim of this study was to determine whether there are any differences between males and females in EDA measurements under the effects of skin hydration. To achieve this, all EDA parameters—skin conductance (SC), skin potential (SP), and skin susceptance (SS)—were simultaneously recorded from both gender groups using a new, non-invasive bioimpedance technique.

## Materials and methods

### Instrumentation

The three-electrode system enables simultaneous measurement of SC, SP, and SS and a system similar to the one described in [24, 25] was used. The system is composed of a small front-end electronic box designed for connection to a data acquisition (DAQ) card that is interfaced to a PC laptop running the LabVIEW software, version 14. The three electrodes were a measuring electrode, a reference electrode, and a current-sink electrode. The current-sink electrode and the measuring electrode provided AC for the SC and SS measurements, at the same time the measuring electrode and the reference electrode were used to measure SP.

### Moisturizer

To hydrate the participants' hands, especially the locations for electrode connections, 0.2 grams of a gel (0.5% KCl with 2% agar) was applied to the chosen skin regions.

### Study protocol

60 (30 male and 30 female) healthy subjects aged between 19 and 43 years from students and staff of the University of Zakho participated in the current investigation. They were involved voluntarily and signed a written informed consent before taking part in the study. All the test subjects were seated in a comfortable chair throughout both experiments in a silent room with the normal room temperature (22–23 °C). In addition, they were instructed to keep calm and avoid bodily movement. Furthermore, both groups were instructed to avoid hand washing and engaging in any physical activities prior to the tests.

To produce mental stress and evoke SCRs, SPRs, and SSRs both groups (males and females) were subjected to four specific stimuli during both experiments. The stimuli were a mathematical task (test subjects were asked to subtract 13 from 100 once for 5 seconds); sound (test subjects were exposed to an acoustic handclap for 1 second); image (test subjects were asked to look at a frightening photo for 3 seconds); and deep breath (test subjects were asked to take a deep breath for 4 seconds). It is important to note that, the order of both the mathematical task and image stimuli were changed in the second experiment to avoid habituation. Moreover, in both experiments, five minutes were allowed for electrode stabilization before measurements started, and there was 60 seconds of relaxation time before and after each stimulus to get the baseline of the measurements.

In the first experiment, EDA measurements were taken from both males and females at the normal skin state without applying any gel on their hands. In the second experiment, both groups were asked to redo the test, but before measurements started 0.2 grams of the moisturizers were applied to all participants' hands in order to hydrate their hands, and then the electrodes were placed over the moisturized areas of the skin (hands).

### Data and statistical analysis

Amplitudes and levels of SC, SP, and SS were computed from EDA waveforms recorded from males and females to analyze the differences between both groups. Levels of SC, SP, and SS were obtained from the average of SCRs, SPRs, and SSRs to four stimuli, respectively. Amplitudes of SC, SP, and SS were calculated from the differences between the response onsets and peaks of SCRs, SPRs, and SSRs, respectively, similar to the procedure illustrated in [24]. In addition, the differences between amplitudes and levels of SC, SP, and SS obtained from both groups were statistically evaluated using the Mann-Whitney U test. The statistical analyses were performed by the Statistical Package for Social Sciences (SPSS).

### Informed consent

Informed consent has been obtained from all individuals included in this study.

### Ethical approval

The protocol has been complied with all relevant national regulations, institutional policies and in accordance with the tenets of the Helsinki Declaration, and has been approved by the authors’ institutional review board or equivalent committee.

## Results

### EDA level

#### Skin conductance level (SCL)

**Fig. 1:**
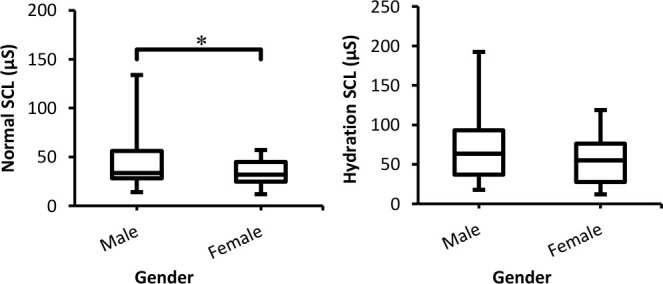
Box-plot with medians, quartiles and the min and max as whiskers shows SCL as a function gender for both normal and hydration conditions. *p = 0.0037.

Data for SCL over 60 (30 males and 30 females) participants as a function of gender for both conditions (normal and hydration) are presented in Figure 1. Inspection of the figure and statistical analysis with the Mann-Whitney U test showed that males produce a higher SCL than females in both conditions. However, with the hydration condition, the differences were reduced to insignificant (*p* =0.087).

#### Skin Potential Level (SPL)

**Fig. 2:**
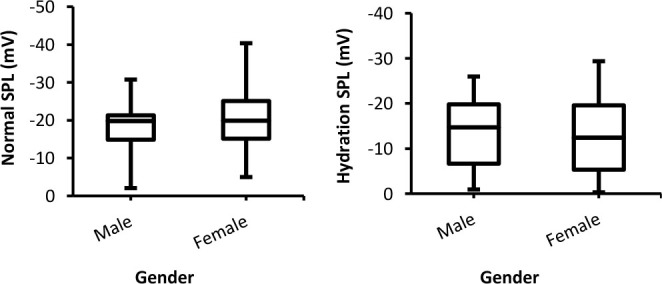
Box-plot with medians, quartiles and the min and max as whiskers shows SPL as a function gender for both normal and hydration conditions.

SPL values recorded from both groups during the normal and hydration experiments are illustrated in Figure 2. The figure depicts a small insignificant (*p* > 0.05) difference between males and females in both conditions. Moreover, at normal condition, females showed a higher (more negative) SPL than males, whereas at the hydration condition, this trend was reversed and males showed slightly (negative) higher SPL than females.

#### Skin Susceptance Level (SSL)

**Fig. 3:**
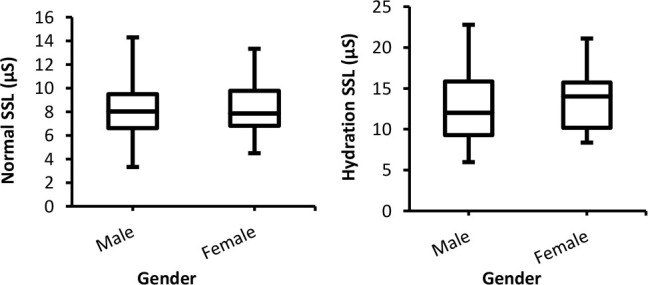
Box-plot with medians, quartiles and the min and max as whiskers shows SSL as a function of gender for both normal and hydration conditions.

Figure 3 presents SSL data for males and females during two various experimental conditions. Inspection of the Figure indicates that, although there are small differences between both groups, the differences were statistically insignificant (*p*>0.05).

### Amplitude of EDA response

#### Amplitude of skin conductance response (SCRs_Amp)

**Fig. 4:**
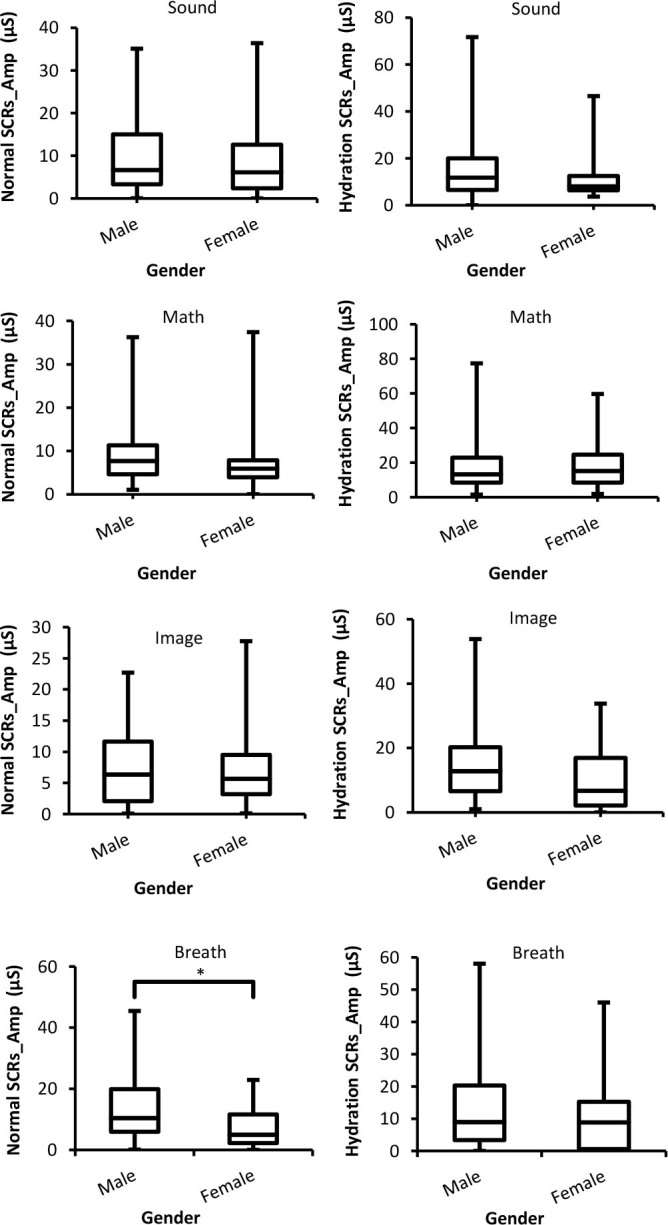
Box-plot with medians, quartiles and the min and max as whiskers shows SCRs_Amp as a function gender for both normal and hydration conditions for all stimuli. *p = 0.008

SCRs_Amp as a function of gender for various stimuli (sound, math, image, and deep breath) and two different experiments are presented in Figure 4. Even though the amplitude of SCRs for males appears slightly higher than for females, the statistical analysis revealed that these differences are not significant (*p*> 0.05) as indicated by the Mann-Whitney U test.

#### Amplitude of skin potential response (SPRs_Amp)

**Fig. 5:**
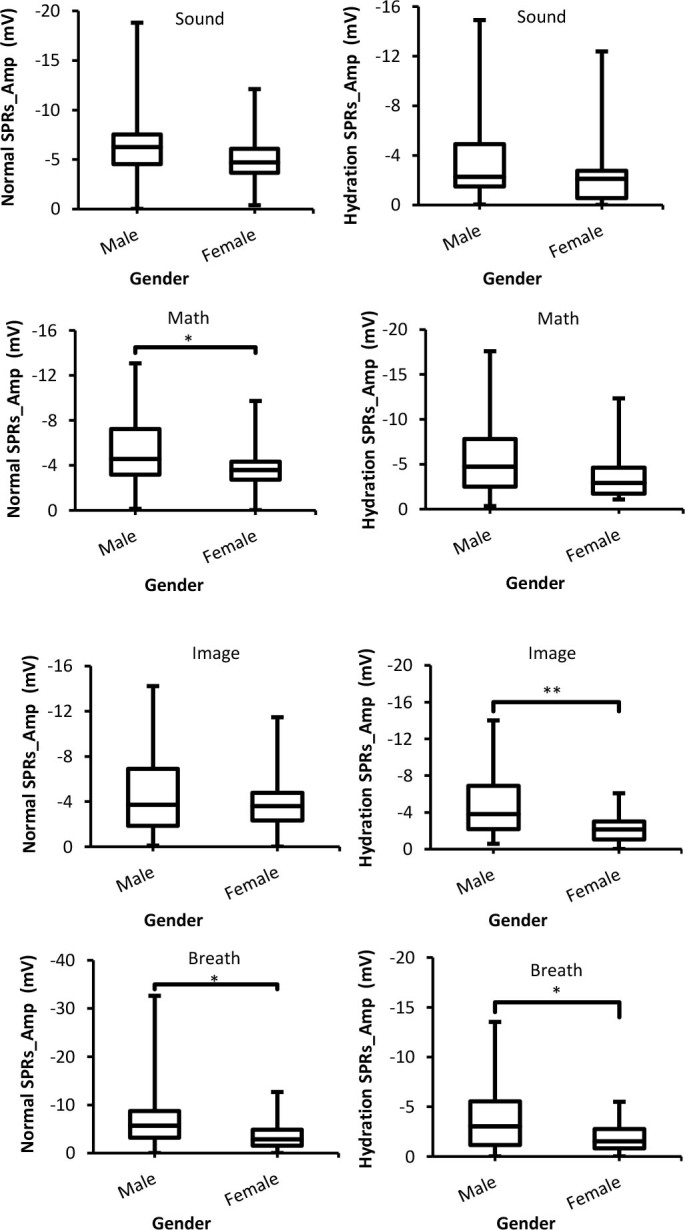
Box-plot with medians, quartiles and the min and max as whiskers shows SPRs_Amp as a function gender for both normal and hydration conditions for all stimuli. *p = 0.02, and **p = 0.001.

Figure 5 shows SPRs_Amp with respect to males and females and two experimental conditions for four external stimuli. It can be seen through the figure that males produce a larger (more negative) SPRs_Amp than females. In addition, the differences between both genders were significant (*p* < 0.05) except for sound stimuli. Furthermore, the same situation (males cause larger SPRs_Amp than females) was seen in the hydration experiment as well.

#### Amplitude of skin susceptance response (SSRs_Amp)

**Fig. 6:**
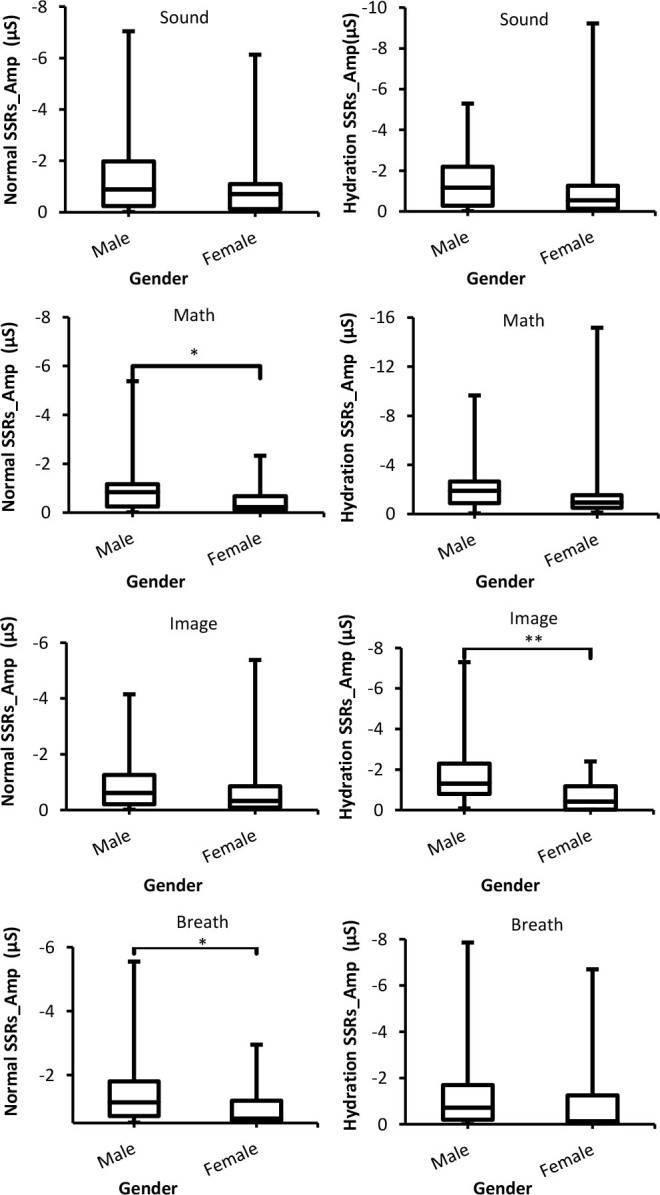
Box-plot with medians, quartiles and the min and max as whiskers shows SSRs_Amp as a function gender for both normal and hydration conditions for all stimuli. *p = 0.02, and, **p = 0.005.

The results of SSRs_Amp obtained from normal and hydration experiments for multiple stimuli as a function of different genders are presented in Figure 6. In both experiments, males showed a higher SSRs_Amp than females, but these differences were not significant (*p* > 0.05) with some stimuli such as sound, math, and breath during the hydration test.

## Discussion

This work represents the first study investigating the gender-related differences in the effects of skin hydration on EDA. The main finding suggests that hydration does not differentially affect the EDA measurements obtained from the two gender groups. Moreover, small gender differences in tonic and phasic EDA parameters during the normal and hydration experiments were almost insignificant (*p*>0.05) as evaluated by the Mann-Whitney U test.

The differences between both genders concerning the three tonic (SCL, SPL, and SSL) parameters were non-significant. Based on the results presented in Figure 1, although in the normal experiment, the differences between males and females were significant, in the hydration experiment the differences between both groups were insignificant (p>0.05). This should mean that hydration had the same effects on the skin resistance (conductance) of males and females. In addition, the values of SC of both groups were increased compared to the normal experiment due to reduction in the skin resistance and an increment in SC [11, 26]. Likewise, the effects of hydration on SPL recorded from both sexes were similar since the differences between them were insignificant (Figure 2). Compared to the normal experiment, the negativity of SPL for both groups was reduced, which might be due to a decrease in epidermal resistance following hydration since epidermal skin acts as a short circuit to the skin surface [21, 27].

Also, hydration does not differentially affect the third tonic parameters (SSL) computed from both males and females. Furthermore, the SSL values of both groups were increased in the hydration experiment due to the increase in the moisture content of their skin [28].

Concerning the phasic EDA component, findings of gender-related differences in the effects of skin hydration were different depending on the EDA parameter and types of external stimuli. There were insignificant differences between SCRs_Amp data of males and females for all stimuli (except breath) in the normal condition experiment, which is in agreement with the results of [4]. In the second experiment, hydration did not lead to significant differences between both genders in SCRs_Amp for all stimuli (sound, math, image, and breath). In addition, SCRs_Amp of both groups were increased in the hydration experiment. This is in contrast to the results of [20], who reported that SCRs_Amp were lowered following skin hydration. Moreover, they illustrated that high hydration produces swelling and a mechanical blockage of the sweat gland in the skin that might impede pore opening and weaken the SCRs_Amp, but this did not happen in this study. This may suggest that no mechanical blockage occurred in the sweat glands. One possible explanation for this finding is that the amount of moisturizer applied (0.2 grams) may not have been enough to hydrate the skin to a large degree, preventing any blockage in the sweat pores.

With regards to SPRs_Amp, there were significant differences between males and females for image and breath stimuli in the hydration experiment. Whereas there were insignificant differences between SPRs_Amp results for both groups at the same condition of the skin. Moreover, males showed a higher (more negative) SPRs_Amp than females in line with the findings of [4, 29]. In the hydration experiment, SPRs_Amp of both genders were lowered. Based on the Edelberg’s model of the internal circuitry of the skin and sweat glands [30], SPRs_Amp is decreased as a result of skin hydration because it prohibits the decrement in resistance of the sweat gland pathway related to a response. Our results of SPRs_Amp are supported by the findings of studies of Fowles and Rosenberry [19], Fowles and Schneider [23], and Garwood et al. [21].

Hydration also increased SSRs_Amp of both males and females compared to normal experiments. In addition, it seems SSRs_Amp of both groups increased (in the negative trend) at the same rate since there were insignificant differences between them. In other words, the increase in SSRs_Amp for both genders in a similar manner is indicative of increasing the electrical capacitance of the skin of the two groups that is proportional to the hydration of the skin [28]. The results of insignificant differences between males and females are supported by the findings of [4], who also observed that the differences between both genders for SSRs_Amp are not significant.

Finally, based on the results presented in this study, males showed a higher reactivity (larger SCRs_Amp, SPRs_Amp, and SSRs_Amp) to stimuli than females in both normal and hydration experiments. This could be because males tend to show a greater amplitude of EDA responses than females under conditions of stimulation [11].

## Limitations

The conclusion is supported by the results, but the study's generalizability might be limited to the specific population (students and staff from one university). Additionally, the moisturizer's composition (0.5% KC1 with 2% agar) might have specific effects not generalizable to other hydrating agents and comparing it with other moisturizers could strengthen the findings.

## Conclusion

Generally, gender-related differences in the effects of skin hydration on EDA especially tonic parameters (SCL, SPL, and SSL) were not observed in this study. However, some differences were noted between males and females in phasic parameters (SPRs_Amp) in response to certain stimuli, which might be attributed to higher reactivity in males than females under stimulation conditions. Moreover, the level and amplitude of EDA signals of both genders were increased during the hydration experiment, likely due to changes in the resistive and capacitive properties of the skin. Hence, it is reasonable to conclude that skin hydration does not contribute to gender-related differences in EDA measurements. This finding is likely significant for both EDA research studies and clinical applications.
